# Changes in R-wave amplitude at implantation are associated with gender and orientation of insertable cardiac monitor: observations from the confirm Rx™ body posture and physical activity study

**DOI:** 10.1186/s12872-022-02752-0

**Published:** 2022-10-08

**Authors:** Matthew Swale, Vincent Paul, Sinny Delacroix, Glenn Young, Luke McSpadden, Kyungmoo Ryu, David Di Fiore, Maria Santos, Isabel Tan, Andre Conradie, MyNgan Duong, Nisha Schwarz, Stephen Worthley, Stephen Pavia

**Affiliations:** 1 The Valley Private Hospital, Mulgrave, VIC Australia; 2 St. John of God Murdoch Hospital, Murdoch, WA Australia; 3 Abbott, Sylmar, CA USA; 4 GenesisCare, Leabrook, 284 Kensington Road, Leabrook, SA Australia; 5 St. Andrew’s Hospital, Adelaide, SA Australia; 6 Friendly Society Private Hospital, Bundaberg, QLD Australia; 7 Perth Mount, Perth, WA Australia; 8 The Wesley Hospital, Auchenflower, QLD Australia

**Keywords:** Arrhythmia, Posture, Gender, Insertable cardiac monitor, R-wave amplitudes, Syncope

## Abstract

**Background:**

Insertable cardiac monitors (ICMs) are small subcutaneously implanted devices that detect changes in R-wave amplitudes (RWAs), effective in arrhythmia-monitoring. Although ICMs have proven to be immensely successful, electrical artefacts are frequent and can lead to misdiagnosis. Thus, there is a growing need to sustain and increase efficacy in detection rates by gaining insight into various patient-specific factors such as body postures and activities.

**Methods:**

RWAs were measured in 15 separate postures, including supine, lying on the right-side (RS) or left-side (LS) and sitting, and two separate ICM orientations, immediately after implantation of Confirm Rx™ ICM in 99 patients.

**Results:**

The patients (53 females and 46 males, mean ages 66.62 ± 14.7 and 66.40 ± 12.25 years, respectively) had attenuated RWAs in RS, LS and sitting by ~ 26.4%, ~ 27.8% and ~ 21.2% respectively, compared to supine. Gender-based analysis indicated RWAs in RS (0.32 mV (0.09–1.03 mV), *p* < 0.0001) and LS (0.37 mV (0.11–1.03 mV), *p* = 0.004) to be significantly attenuated compared to supine (0.52 mV (0.20–1.03 mV) for female participants. Similar attenuation was not evident for male participants. Further, parasternally oriented ICMs (*n* = 44), attenuated RWAs in RS (0.37 mV(0.09–1.03 mV), *p* = 0.05) and LS (0.34 mV (0.11–1.03 mV), *p* = 0.02) compared to supine (0.48 mV (0.09–1.03 mV). Similar differences were not observed in participants with ICMs in the 45°-relative-to-sternum (*n* = 46) orientation. When assessing the combined effect of gender and ICM orientation, female participants demonstrated plausible attenuation in RWAs for RS and LS postures compared to supine, an effect not observed in male participants.

**Conclusion:**

This is the first known study depicting the effects on RWA due to body postures and activities immediately post-implantation with an overt impact by gender and orientation of ICM. Future work assessing the cause of gender-based differences in RWAs may be critical.

*Trial registration:* Clinical Trials, NCT03803969. Registered 15 January 2019 – Retrospectively registered, https://clinicaltrials.gov/NCT03803969

## Background

Abnormalities of cardiac rhythm such as atrial fibrillation (AF), supraventricular tachycardias, bradycardia and ventricular arrythmias, are associated with significant morbidity and economic burden. In scenarios where an arrythmia is difficult-to-diagnose, cardiac rhythm monitoring could prove to be beneficial for diagnostic assessment and therapeutic intervention especially in patients who present with palpitations, syncope or cryptogenic stroke [1–3]. Electrocardiography (ECG) serves as the gold standard for non-invasive monitoring of cardiac rhythm and conduction disturbances. However, the frequently utilised 12-lead ECG provides seconds of cardiac rhythm monitoring which is inadequate largely due to lack of symptoms during ECG assessment. Although, other non-invasive systems such as external loop recorders, wireless patch monitors and mobile cardiac telemetry system are useful in standard clinical practice, they can be bulky and limited in providing extended cardiac rhythm surveillance yielding low patient compliance or even more concerningly, low detection rates for silent arrythmias [4].

Insertable cardiac monitors (ICMs) are currently established as significant counterparts in the diagnosis of arrythmias with greater than 78% detection rates, thus providing a correlation between heart rhythm and cardiac symptoms [5, 6]. With regards to syncope and cryptogenic stroke, landmark studies including CRYSTAL-AF [6], PICTURE[7, 8] and others [9–12] have now consistently shown the safety and efficacy of ICMs in the diagnosis of arrythmias or AF with a detection rate of ~10–30% compared to non-ICM control arms. ICMs have also been useful as an exclusion tool in the diagnosis of arrythmias thereby enhancing standard-of-care [13, 14]. The FRESH study reported a definite cause of syncope in 46% of the ICM group compared to 5% in the conventional group (suggestive of greater than 40% additional benefit with an ICM). Reinforcing the study’s results, no differences were reported in quality of life between the two treatment arms possibly due to less cardiac testing in the ICM group [14].

Despite the past decade’s advancements in diagnosis of arrythmias and AF by ICMs, some limitations such as sub-optimal ECG, short memory storage and risk of procedural complications, have hindered diagnosis in patients presenting with syncope or cryptogenic stroke. The main limitation being the inability to obtain clear and accurate subcutaneous ECG in prolonged monitoring leading to artefacts and non-diagnostic interrogations. A QRS or R wave under-sensing and oversensing may reflect false asystole and false high ventricular rate episodes, respectively [15, 16] and thus, mislead diagnosis. Although, recommendations for optimal positioning and procedural methodologies for implanting ICM have been investigated and provided some success, patient-specific features and complexity of cardiac pathologies have confounded further progress, particularly for arrythmias/AF arising from syncope and cryptogenic stroke [17, 18].

This study was performed to address one such cause of limitation in ICM-based arrythmia detection, patient body postures and movements. Body postures have increasingly gained attention following findings from a comparative study of surface and subcutaneous ECGs by Bellardine Black et al*.* (2010) [19]. It was reported that in an assessment of 12 controlled body postures and movements, surface ECGs were an adequate surrogate of subcutaneous ECGs during resting and isometric myopotential noise conditions. However, in situations of patient movement, surface ECGs was not representative of subcutaneous ECG owing to significantly worse signal to noise ratio. This study also demonstrated intra-patient variability in R-wave amplitudes (RWAs) due to common body postures in patients clinically indicated for an ICM. Here, we present findings on fifteen separate controlled body postural activities immediately following implantation with the Confirm Rx™ ICM (Abbott Medical Devices, Australia).

## Methods

### Study participants

**Fig. 1:**
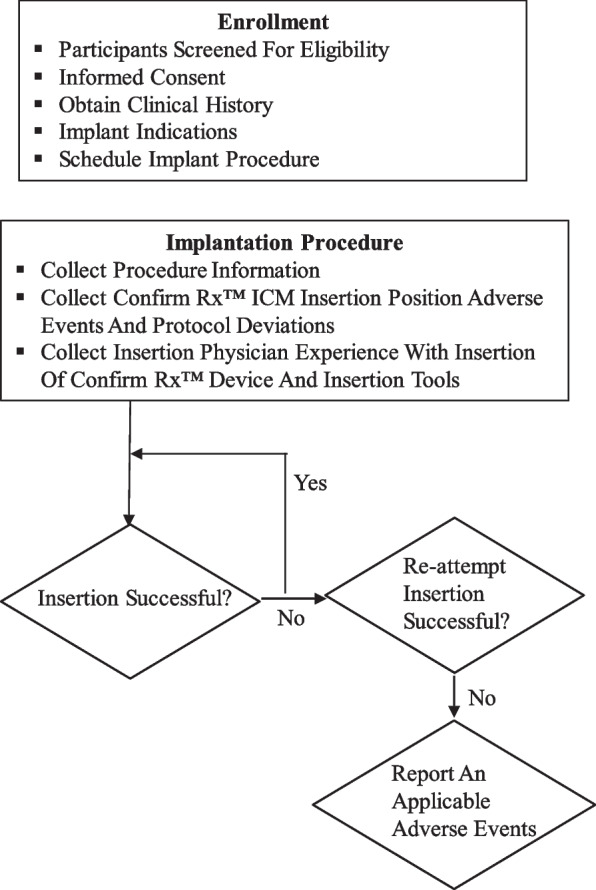
Flowchart depicts Confirm Rx ™ Insertable Cardiac Monitor (ICM) study plan. Details on eligibility criteria provided in methods. ICM-Insertable Cardiac Monitor

The study was designed as a prospective, single arm, unblinded, non-randomised, open-label, multi-centre, post-market clinical investigation of 100 patients who were clinically indicated an implantable cardiac monitor with participants enrolled at 6 centres across Australia (Fig. 1 depicts study plan). All participants were implanted with the Confirm Rx ICM either parallel- or 45°relative to sternum over the 4th intercostal space. Patients with a clinically indication for an ICM and scheduled to receive a Confirm Rx ICM were eligible if they were greater than 18 years of age, had no previous cardiac implantable electronic device (CIED) and provided informed consent to participate in the study. One participant was withdrawn since the patient had received a pacemaker prior to Confirm Rx ICM implantation. The Clinical Investigation Plan and informed consent forms were reviewed and approved by the central and local human research ethics and governance committees. The trial was designed in accordance with the Declaration of Helsinki and registered with ClinicalTrials.gov (Trial ID: NCT03803969).

### Confirm Rx ICM device specifications

In Australia, the Confirm Rx ICM is indicated for monitoring and diagnostic evaluation of patients experiencing unexplained symptoms such as dizziness, palpitations, chest pain, syncope, and shortness of breath, as well as patients who are at risk of cardiac arrhythmias, have previously been diagnosed or are at increased risk of arrythmias such as AF since 2017. Confirm Rx ICM Model DM3500 is a minimally invasive, insertable diagnostic monitoring device with subcutaneous electrodes, looping memory and automatic as well as patient-activated electrogram (EGM) storage capability. It is an improved model of its predicate device (Confirm DM2102, St. Jude Medical) and includes a downsized hardware, Bluetooth communication, MRI conditional labelling with identical arrhythmia detection algorithms as St. Jude Medical Confirm Rx™ DM2102. Specific features include patient-initiated triggering of EGM storage using the myMerlin™ patient app for mobile devices.

### Study procedure and data collection

Prior to commencement of procedure, patient baseline characteristics and cardiac history were documented. An electrocardiogram and echocardiogram were also performed at baseline to ascertain cardiac rhythm, function and indication for ICM. Within one hour following implantation of ICM and device programming, participants were requested to perform fifteen different body postures and activities for at least 30 s with one repetition (all body posture and activities have been defined in (Table 1). RWAs were recorded using the Merlin™ programmer (Model 3330, Abbott Medical). Following ICM implantation and programming, adjusted RWAs were measured twice for each participant at each of the fifteen postural activities and, the mean of the two amplitudes was reported as the measured RWA for that data point.

**Table 1:** Describes fifteen body postural activities performed immediately following implantation

Postural activity	Definition
Supine	Participant lies on back
Right side	Participant lies on right side
Left side	Participant lies on left side
Sitting	Participant is sitting
Standing	Participant is standing
Isometric push	While sitting, subject presses both palms together (in an opposing manner) and pushes as hard as possible
Isometric pull	While sitting, subject locks fingers together and pulls sideways and outwards as hard as possible
Ballottement	The implanted device is moved back and forth (3 times per second) along ICM’s length
Chest thumping	The participant’s chest is tapped quickly (three times per second) on the ConfirmRx™ ICM
Chest pressure	Brisk pressure is applied on the device at three levels 1cms apart if possible (upper/middle/lower)
Arm flaps	Participant extends the arms fully at side, raises them outward and lowers them in an alternating fashion (“butterfly manoeuvre”)
Left arm handshake	The subject shakes hands strongly using left arm with a study personnel
Brisk hall walks	Patient walks with intent to get heart rate to 90 beats per minute (bpm)

### Statistical analysis

Statistical analysis was performed using GraphPad Prism version 8.0.0 for Windows, GraphPad Software (San Diego, California USA, www.graphpad.com). Plots have been represented as box-and-whiskers plot with the whiskers depicting minimum and maximum values. *P* values < 0.05 were considered significant. Values have been expressed as median with range as minimum RWA and maximum RWA, unless otherwise stated. Repeated measures analysis of variance was used to evaluate RWAs and interaction of effects on body postures and movements. When normality could not be met, Kruskal–Wallis tests was performed.

## Results

### Participant baseline characteristics

Over the enrolment period 99 patients clinically indicated for an ICM implantation procedure consented to participate in this study. Majority of the patients (66.7%) presented with syncope as the main indication followed by dizziness (31.3%) and palpitations (19.2%) in the remaining patients. The study included 53 female and 46 male participants with a mean age of 66.6 ± 14.7yrs for females and 66.4 ± 12.3yrs for males. The mean BMI was 27.1 ± 5.4 kg/m^2^ and 27.4 ± 5.1 kg/m^2^ for female and male participants, respectively.

### Procedural features

Procedural characteristics have been summarised in (Table 2). All procedures were performed under local anaesthesia and/or conscious sedation with 21.4% of patients receiving intravenous pre-procedural antibiotics. The average length of time taken to implant Confirm Rx was ~6 min and overall technical success was 100%. No repositioning was required for 93.7% of procedures. When repositioning was necessary (6.1%), a single reattempt resulted in adequate RWAs of  ≥0.2 mV which was sustained in 90–95% participants when measuring RWAs for the separate postures. The Confirm Rx ICM was implanted either 45° relative to the sternum over the 4^th^ intercostal space in 51.1% of patients or parallel to the sternum in 48.8% of patients. Adhesive strips were the main method for wound closure (45.5%) followed by sutures (42.4%).

**Table 2:** Patient cohort characteristics

Total *n* = 99	Female	Male
Number of participants	53	46
Age (mean ± SD)	66.62 ± 14.73	66.40 ± 12.25
BMI (mean kg/m^2^ ± SD)	27.10 ± 5.38	27.40 ± 5.10
Co-morbidities		
CAD	9.09	12.12
MI	3.03	3.03
Angina	2.02	1.01
CABG	2.02	2.02
PTCA	6.06	6.06
Hypertension	25.25	23.23
Hypercholesterolemia	17.17	21.21
Diabetes	4.04	5.05

^#^Values are shown as percentages of the total number of participants enrolled, unless otherwise stated. BMI, body mass index; CAD, coronary artery disease; MI, myocardial infarction; CABG, coronary artery bypass grafting; PTCA, percutaneous transluminal coronary angioplasty; SD, standard deviation.

### Effect of postural activities on R-wave amplitude

**Fig. 2:**
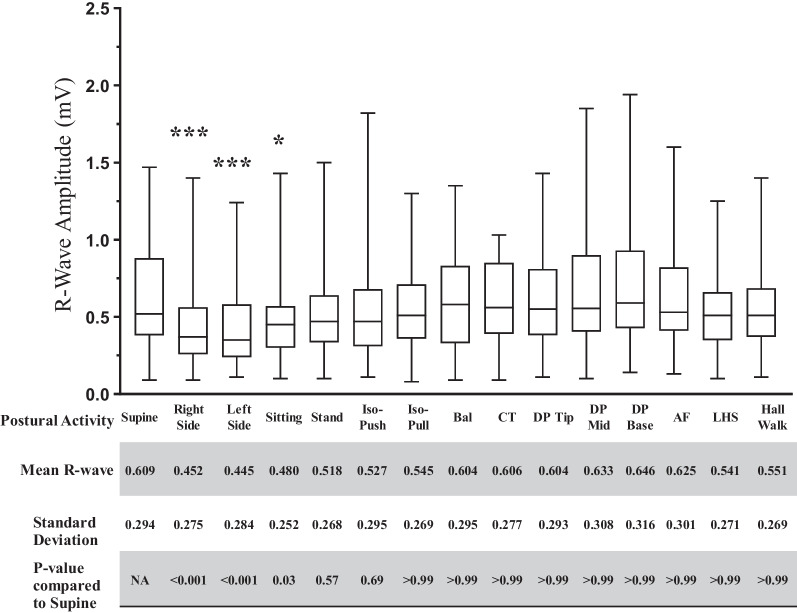
*R*-wave amplitudes detected across all postural activities immediately following implant. Participant RWAs were assessed across 15 separate postures immediately following insertion of Confirm Rx™ ICM. On comparison to the Supine posture, lying on Right Side or Left side and Sitting were found to be significantly different in RWAs. Values for RWAs have been depicted as median and values range from minimum to maximum (*n* = 99). Significance was determined by Kruskal–Wallis test with p-value of < 0.05 considered significant. *** *p*-value of <0.001, *p-value of <0.05. Stand- Standing, Iso-Push- Isometric Push, Iso-Pull- Isometric Pull, Bal-Ballottement, CT- Chest Thumping, DP-Tip/Mid/Base- Device Pressure/Pressure on ICM at the tip, middle or lower part, AF- Arm Flaps, LHS- Left Arm Handshakes

Within one hour following implantation, patients were assessed for RWAs in all fifteen postural activities and RWA was measured twice for each posture and activity to obtain the mean measurement for each data point. Data was assessed for normality prior to performing statistical tests. Both, median and mean RWAs are shown in (Fig. 2). To understand the effect of various body positions and movements on RWA variability, we compared RWAs’ detected at all postural activities to supine posture. RWAs detected by the Confirm Rx ICM showed evident inter-participant variability at each postural activity with overall variability (median 0.51 mV, range 0.84–1.94 mV) ranging from +5.5% to −27.8% (mean difference for all postures relative to supine was 9.2% ± 10.9%) in RWAs when compared to the supine position. To determine specific postural activities with significant RWA variations individual significance tests were performed which revealed three positions, lying on right side (RS), lying on left side (LS) and sitting having the most significant effect on RWAs when compared to supine with mean ± SD for RWAs as 0.45 mV (± 0.28 mV), 0.45 mV(± 0.28 mV) and 0.48 mV(± 0.25 mV) vs. 0.61 mV (± 0.21 mV), respectively. Thus, in comparison to supine, RS, LS and sitting postures showed a reduction in RWA of 26.4%, 27.8% and 21.2% respectively.

### Effect of gender on R-wave amplitudes

**Fig. 3:**
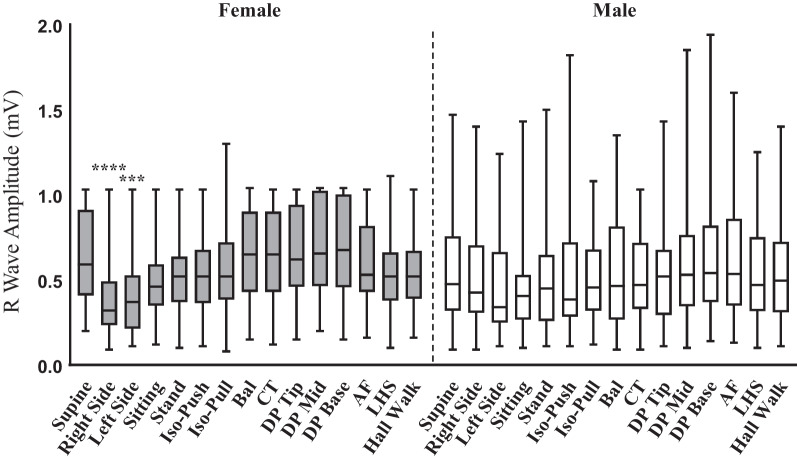
*R*-wave amplitude showed significant association to lying on right side-and left side compared to supine posture in female participants. RWAs were plotted as Female (*n* = 53) vs Male (*n* = 46) participants. Values for *R*-waves have been depicted as median and values range from minimum to maximum. Significance was determined by Kruskal–Wallis test with *p*-value as <0.05 considered significant. **** *P*-value is <0.0001, *** *p*-value is <0.001. Stand- Standing, Iso-Push- Isometric Push, Iso-Pull- Isometric Pull, Bal-Ballottement, CT- Chest Thumping, DP-Tip/Mid/Base- Device Pressure/Pressure on ICM at the tip, middle or lower part, AF- Arm Flaps, LHS- Left Arm Handshakes

All fifteen postural activities separated by gender were analysed to assess the effect gender had on variabilities observed in RWAs (Fig. 3). The first observation was the overall pattern for RWAs between the two cohorts, where male participants illustrated greater inter-participant and inter-postural variability compared to females (median RWA with range for females was 0.52 mV(0.08–1.30 mV) vs. 0.46 mV (0.09–1.94 mV) for males). Secondly, in female participants RS (0.32 mV (0.09–1.03 mV), *p*-value < 0.0001) and LS (0.37 mV (0.11–1.03 mV), *p*-value = 0.004) postural activities were significantly different to supine (0.52 mV (0.20–1.03 mV)). Lastly, gender-based analysis of the third postural activity of interest, sitting, was significant only in females (median RWA for sitting 0.46 mV (0.12–1.3 mV) vs median RWA for supine 0.52 mV (0.20–1.30, *p*-value = 0.004).

### Effect of confirm Rx ICM implant orientation on R-wave amplitudes

**Fig. 4:**
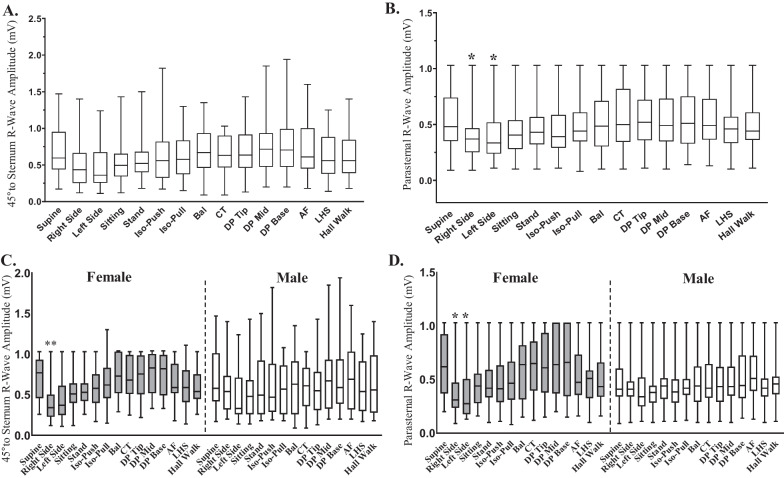
Position of ICM implant affected R-wave amplitude detected for female participants immediately following implant. RWAs for the two anatomical positions **A.** 45°relative to sternum (*n* = 46) and **B.** parallel to sternum (*n* = 44), selected for implanting Confirm Rx™ ICM. Parasternal positioning of ICM depicted differences in RS and LS postures compared to supine. Effect of gender was observed for RS and, RS and LS postures compared to Supine was observed for **C**. 45°relative to sternum (female *n* = 23 vs male *n* = 23) and **D.** parallel to sternum (female *n* = 26 vs male *n* = 18), respectively. Values for RWAs have been depicted as median and values range as minimum to maximum. Significance was determined by Kruskal–Wallis test or Mixed-effects Analysis with *p*-value as < 0.05 considered significant. *P-value is <0.05. RS = lying on Right Side, LS = lying on Left Side, Stand- Standing, Iso-Push- Isometric Push, Iso-Pull- Isometric Pull, Bal-Ballottement, CT- Chest Thumping, DP-Tip/Mid/Base- Device Pressure/Pressure on ICM at the tip, middle or lower part, AF- Arm Flaps, LHS- Left Arm Handshakes

Participants implanted with Confirm Rx ICM in either the 45° to sternum or parasternal site (Fig. 4A , B) showed greater inter-participant variability in RWAs at 45° to sternum (0.55 mV (0.09–1.94 mV)) compared to parasternally implanted ICMs (0.44 mV (0.08–1.03 mV)). Further, when all postures were compared to supine, an estimate 7.8% decreased sensing of overall RWA was observed for 45° to sternum orientation compared to ~ 11.0% reduction in overall RWAs’ for parasternally oriented ICMs, suggestive that 45° to sternum could be a preferred choice for future implantations.

When assessing the separate postural activities, we observed that if Confirm Rx was located 45° to sternum, no significant differences in RWAs were detected between any of the postural activities (Table 3). However, this was not the case for ICMs positioned parasternally, where RS and LS postures depicted plausible reductions in RWAs of ~28.4% (0.37 mV(0.09–1.03 mV), *p*-value = 0.05) and ~29.9% (0.34 mV(0.11–1.03 mV), *p*-value = 0.02) respectively compared to supine (0.48 mV(0.09–1.03 mV)).

**Table 3:** Procedural characteristics

Total *n* = 99	
Success (%)	100%#
Procedure time (mean in minutes ± SD)	5.88 ± 3.77
Periprocedural antibiotics	21.4%
*ConfirmRx™ ICMImplant Position*	
Parasternal	49.89%
45° to sternum	51.11%

^#^Values are shown as percentages of the total number of participants enrolled, unless otherwise stated

To evaluate the effect of gender on implantation site, we first assessed 45° to sternum based on gender for all postural activities (Fig. 4C). On comparison to supine, no significant changes occurred in RWAs for Confirm Rx devices implanted 45° to the sternum for male participants however, RWAs detected at RS were significantly reduced by ~42.0% (RS—0.34 mV(0.12–1.03 mV) vs. Supine-0.77 (0.26–1.03 mV), *p*-value = 0.0023) in female participants for the same. In participants with parasternally implanted ICMs the RWAs detected for RS and LS were significantly different to supine (Fig. 4D). We observed ~36.9% and ~38.2% reduction in RWAs in the RS (0.31 (0.09–1.03 mV), *p*-value = 0.023) and LS (0.28 mV (0.13–1.03 mV)) postures respectively compared to supine (0.62 (0.20–1.03 mV), *p*-value = 0.022).

## Discussion

This is the first study describing the impact of body postures and activities on RWA immediately following implantation of an ICM. The main findings of this study were firstly, 100% of the participants were successfully implanted with Confirm Rx ICMs, following which RWAs were recorded for all fifteen body postures and physical activities within one hour of implantation. Secondly, there was a considerable effect on RWAs in different body positions compared to supine. The study showed that lying on the RS or LS reduced RWAs by ~28%. Thirdly, the variability in RWAs were more evident for male participants, particularly in 45° to sternum orientation for Confirm Rx ICMs indicative of gender-specific factors which may impact RWA sensing. Further, body mass index (BMI) weakly and negatively correlated with RWAs at different postures and activities [median (range) *r*^2^ = −0.36 (−0.42 to −0.27)] suggestive of low-to-negligible confounding effect of BMI in this study.

The results from this study can be put into context with an understanding of the vast literature emphasising the importance of continuous cardiac rhythm monitoring for detection of arrythmias in syncope or cryptogenic stroke. Seminal studies such as RAST (Randomized Assessment of Syncope Trial) [20], PICTURE (Place of Reveal in the Care Pathway and Treatment of Patients with Unexplained Recurrent Syncope)[7], and CRYSTAL-AF [6] trials revealed that ICMs were capable and more successful in detecting AF in patients with syncope (30–36% increased detection rate over a period of ~ 10 months) and cryptogenic stroke (sixfold increased detection by 12 months) than conventional monitoring. Despite advances in ease-of-implant, remote-monitoring and data collection capabilities, ICMs are not devoid of RWA sensing irregularities [21]. These sensing insufficiencies and related inaccuracies in rhythm detection can delay the diagnosis of arrythmias in syncope and cryptogenic stroke as demonstrated in several small studies reporting the effect of body postures, activity and site of implantation on signal quality received from external or subcutaneous ECG [15, 19, 22–24]. The study by Bellardine Black *et.al*., published in 2010 [19], compared the consistency in signals received simultaneously from an implantable loop recorder and surface ECGs. The study revealed that the RWAs received from the two modalities highly correlated in resting (equivalent to supine) body positions (*r*^2^ = 0.96) and the correlation reduced to 0.82–0.93 during postural activities that exhibited motion artefacts such as ballottement or chest thumping. An important point of difference in comparison to our study was that all participants were implanted with Reveal® Plus implantable loop recorder, an older model to Reveal LINQ™ and Confirm Rx and, for a minimum of 4 weeks prior to commencement of the study. Although this study provided crucial insights on the effects of body postures/activities, it did not assess the immediate effects of body posture and physical activities on RWAs in patients receiving ICMs. Thus, the results from our study highlight the potential impact of RWA sensing immediately following implantation, providing foundation for additional patient-specific assessments (such as walking, sleeping positions) for setting optimal RWA thresholds.

An additional implication from our study was the preferences for site of ICM implantation. ICM implantation site has shown to affect an ICM’s arrythmia diagnosis rates where as much as twofold under-sensing of R-wave have been illustrated, with the 45°to sternum being the preferred site of implantation [25, 26]. It is, however, important to note that none of these trials documented subcutaneous ECG immediately following implantation as a requisite for assessing efficacy of the ICM, either in combination with specific body postures/activities or site of implantation. Findings from our study are complimentary to current literature, reinforcing 45° to sternum as the likely site for achieving optimal RWA sensing. Although there are other studies such as the BIOSTREAM-ICM (Trial ID: NCT04075084) observational study, occurring in parallel assessing the real-world impact of Biotronik’s BIOMONITOR III with regards to safety and efficacy in a variety of device- and patient-specific aspects over a period of three years, the affects immediately following implantation may not be a primary assessment in these studies.

The final implication of the findings in this study were that female participants implanted with the ICMs observed a reduction in RWAs, particularly in the RS and LS positions compared to supine. The efficacy of ICMs in relation to gender has been investigated in a sub-analysis from the PICTURE study [8]. The sub-study demonstrated that in a cohort of 570 participants (54% females), gender was relevant both in clinical evaluation and in the rate of syncopic episode recurrence and in the subsequent treatment, but not relevant for diagnostic yield of the ICM. The results from the PICTURE sub-study contrast with our findings with respect to gender-related sensing from ICMs which could be due to the study cohort and design being distinct to our study. Further, we are aware of the hypothesis that longer follow-up period from implantation to data collection may result in an increased probability of detecting arrythmia, a concept defined in the PICTURE sub-study. This may be due to a *stabilising period* required for the ICM to perform optimally and can be explained by the initial inflammation and induration of the implant site that impacts tissue contact with the device however, this resolves over time and improves the sensing capability of ICMs, a finding supported by Nico Reinsch *et. al.,* published in 2022 [27]. Despite the contradicting outcome from PICTURE sub-study, a recent study comparing RWAs between genders at longer follow-up of ~204 days depicted lower RWAs for female participants, thus supporting the significant differences observed between the genders in this study [28].

The limitations of the study include the relatively small sample size and the lack of a control group such as data from other ICMs or RWAs at other timepoints (such as 24 h or 1-week post-implantation) which would have provided additional strength to the findings of this study. That said, we believe that to improve sensitivity of detection and reduce noise-to-signal ratio, optimising RWA detection at implant will be key in delineating true event rates for arrythmias. Although, similar studies assessing differences in RWAs at implantation for different postures/activities is lacking, recent evidence of similar observations whilst assessing efficacy of ICM showed variations in RWAs at implantation within patients and between genders [27, 28].

## Conclusion

The findings from our study suggests that the 45° to sternum site for ICM implantation may be more favourable than parasternal implants, providing the least variability irrespective of gender. It could be proposed that in the 45° to sternum position the device was located adequately further away from the heart irrespective of the amount of breast tissue between the heart and the device, and, in the parasternal position the depth of breast tissue could vary individually presenting a factor for increased variability when the device is implanted in this location. The gender differences observed in the study are noteworthy and may provide a foundation for better algorithm development, optimal implantation procedures and thereby efficient event detections.

## Data Availability

The datasets generated during and/or analysed during the current study are not publicly available due to confidentiality agreement but are available from the corresponding author on reasonable request.

## Abbreviations

AF: Arm flaps
Bal: Ballottement of device
CT: Chest thumping
DP-Tip/Mid/Base: Device pressure/pressure on ICM at the tip, middle or lower part
ICD: Implantable cardioverter defibrillator
ICM: Insertable cardiac monitors
Iso-Pull: Isometric pull
Iso-Push: Isometric push
LHS: Left arm handshakes
PCM: Pacemaker
RWA: R-wave amplitudes
